# Machine learning and SHAP value interpretation for predicting cardiovascular disease risk in patients with diabetes using dietary antioxidants

**DOI:** 10.3389/fnut.2025.1612369

**Published:** 2025-07-16

**Authors:** Xinyu Zhang, Sen Lin, Qingling Zeng, Lisheng Peng, Chaoguang Yan

**Affiliations:** ^1^The Fourth Clinical Medical College of Guangzhou University of Chinese Medicine, Shenzhen, Guangdong, China; ^2^School of Pharmaceutical Sciences, Guangzhou University of Chinese Medicine, Guangzhou, Guangdong, China; ^3^Shenzhen Traditional Chinese Medicine Hospital, Shenzhen, Guangdong, China; ^4^Weifang Traditional Chinese Hospital, Weifang, Shandong, China

**Keywords:** machine learning, SHAP, diabetes, cardiovascular disease, dietary antioxidants

## Abstract

**Objective:**

This study aims to develop and validate a machine learning model that integrates dietary antioxidants to predict cardiovascular disease (CVD) risk in diabetic patients. By analyzing the contributions of key antioxidants using SHAP values, the study offers evidence-based insights and dietary recommendations to improve cardiovascular health in diabetic individuals.

**Methods:**

This study leveraged data from the U.S. National Health and Nutrition Examination Survey (NHANES) to develop predictive models incorporating antioxidant-related variables—including vitamins, minerals, and polyphenols—alongside demographic, lifestyle, and health status factors. Data preprocessing involved collinearity removal, standardization, and class imbalance correction. Multiple machine learning models were developed and evaluated using the mlr3 framework, with benchmark testing performed to compare predictive performance. Feature importance in the best-performing model was interpreted using SHapley Additive exPlanations (SHAP).

**Results:**

This study utilized data from 1,356 individuals with diabetes from NHANES, including 332 with comorbid CVD. After removing collinear variables, 27 dietary antioxidant features and 13 baseline covariates were retained. Among all models, XGBoost demonstrated the best predictive performance, with an accuracy of 87.4%, an error rate of 12.6%, and both AUC and PRC values of 0.949. SHAP analysis highlighted Daidzein, magnesium (Mg), epigallocatechin-3-gallate (EGCG), pelargonidin, vitamin A, and theaflavin 3′-gallate as the most influential predictors.

**Conclusion:**

XGBoost exhibited the highest predictive performance for cardiovascular disease risk in diabetic patients. SHAP analysis underscored the prominent contribution of dietary antioxidants, with Daidzein and Mg emerging as the most influential predictors.

## Introduction

Diabetes mellitus (DM) has emerged as one of the most prevalent and serious chronic diseases (1, 2), with patients facing a significantly elevated risk of cardiovascular disease (CVD), which remains the leading cause of mortality in this population (3–5). The mechanisms underlying diabetes-associated cardiovascular disease involve oxidative stress, inflammatory responses, metabolic disturbances, mitochondrial dysfunction, accumulation of advanced glycation end products (AGEs), insulin signaling abnormalities, endoplasmic reticulum stress, and cardiomyocyte death (6, 7). Epidemiological studies indicate that individuals with diabetes have a higher risk of CVD compared to non-diabetic individuals (8). The Framingham Heart Study demonstrated that the attributable risk of CVD due to diabetes increased from 5.4% during 1952–1974 to 8.7% in 1975–1998 (9). Haffner et al. further conducted a 7-year follow-up study on cardiovascular mortality, reporting a mortality rate of 15.4% among diabetic patients without a history of myocardial infarction (MI) and 42.0% among those with MI, compared to 2.1 and 15.9%, respectively, in non-diabetic individuals (10). A 10-year follow-up study by van Hateren et al. also showed that the risk of CVD-related mortality in diabetic patients increased annually (11). Given the substantial clinical burden of CVD complications in diabetic patients, integrated management of diabetes and CVD has become a major focus. Central to this management is effective blood glucose control. While intensive glucose control has proven beneficial in preventing microvascular complications and CVD in type 1 diabetes (12, 13), its role in reducing cardiovascular risk in type 2 diabetes mellitus remains contentious (14–16). Consequently, preventing macrovascular complications requires a comprehensive approach, addressing multiple risk factors such as blood glucose management, a healthy diet, smoking cessation, regular physical activity, blood pressure control, and treatment of dyslipidemia (17, 18).

In recent years, dietary factors, particularly the potential cardiovascular protective effects of dietary antioxidants, have garnered increasing attention (19, 20). Antioxidants in the diet, such as flavonoids, vitamins, and polyphenols, may lower the risk of CVD in diabetic patients by reducing oxidative stress, exerting anti-inflammatory effects, improving vascular function, and regulating metabolic processes. Due to differences in mechanisms of action, metabolism, and bioavailability among antioxidants (21, 22), identifying the most protective compounds may inform more targeted dietary interventions for individuals with diabetes.

This study utilizes data from the National Health and Nutrition Examination Survey (NHANES) and applies machine learning (ML) methods to investigate the potential relationship between dietary antioxidant intake and cardiovascular disease in diabetic patients. Compared to traditional statistical approaches, machine learning techniques are better equipped to manage large, complex datasets and identify intricate relationships among health features, thus enabling more accurate predictions of disease risk (23). To ensure model reliability, we conducted benchmark testing to compare different models and employed SHapley Additive exPlanations (SHAP) values to enhance model interpretability, highlighting the specific contributions of various dietary antioxidants in disease prediction. While previous studies have investigated the cardiovascular effects of individual antioxidants, total antioxidant intake, or antioxidant scores (24–26), they predominantly relied on traditional statistical methods, limiting the ability to assess the relative importance of each antioxidant in disease risk. This study innovates by integrating machine learning with SHAP analysis, enhancing prediction accuracy and precisely quantifying the independent contribution of each antioxidant to cardiovascular disease risk, offering valuable insights for personalized nutrition interventions and risk stratification.

## Participants and methods

### Participants

**Figure 1:**
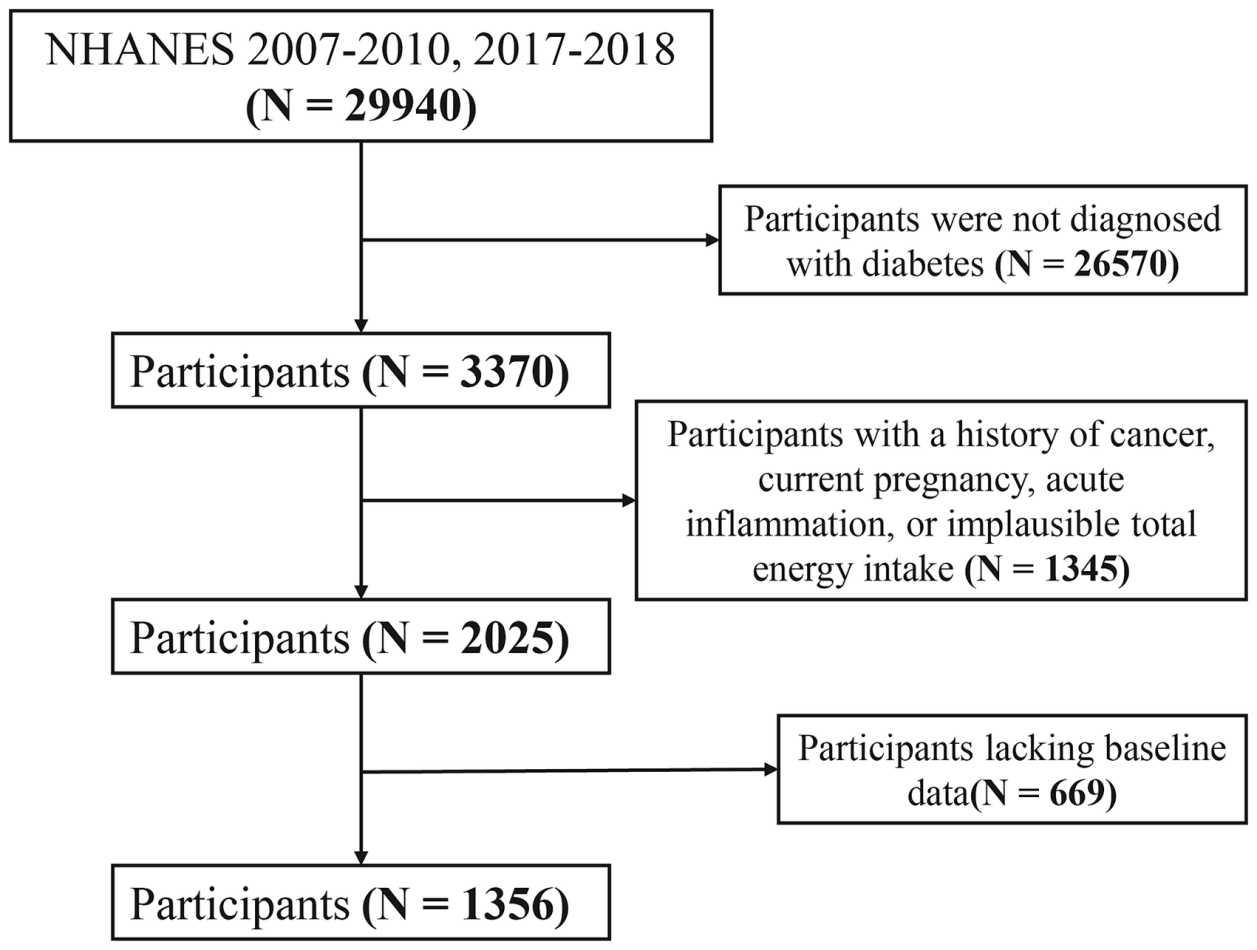
Flowchart of participant selection process.

The National Health and Nutrition Examination Survey (NHANES), administered by the U.S. Centers for Disease Control and Prevention (CDC), collects nationally representative data on health, nutrition, and risk factors through interviews, physical examinations, and laboratory assessments. This study analyzed data from NHANES 2007–2010 and 2017–2018, including participants with complete dietary antioxidant intake data and clearly defined diagnoses of diabetes and CVD. Exclusion criteria included missing baseline data, a history of cancer, pregnancy, CRP levels >10 mg/L (indicative of acute inflammation), and implausible total energy intake (men: <800 or >4,200 kcal/day; women: <500 or >3,500 kcal/day) to reduce confounding. The participant selection flow is presented in Figure 1.

### Dietary antioxidant intake

This study utilized data on the intake of 44 dietary antioxidants (including vitamins, minerals, and polyphenols) from the NHANES dataset. Participants completed two 24-h dietary recall interviews at the mobile examination center, spaced 3 to 10 days apart. The average daily intake of dietary antioxidants was calculated based on these interviews. All dietary data were processed through the USDA’s Food and Nutrient Database for Dietary Studies (FNDDS) and combined with the USDA’s 2007–2010 and 2017–2018 Flavonoid Value Database to ensure accurate calculation of dietary antioxidant intake.

### Diagnosis of CVD and diabetes

CVD diagnosis was based on self-reported physician diagnoses of congestive heart failure, coronary heart disease, angina, myocardial infarction, or stroke. Diabetes diagnosis was determined by self-reported physician diagnosis or meeting at least one of the following criteria: glycated hemoglobin (HbA1c) ≥ 6.5%, fasting plasma glucose (FPG) ≥ 7.0 mmol/L, 2-h plasma glucose in an oral glucose tolerance test (OGTT) ≥ 11.1 mmol/L, or current use of antihyperglycemic medication.

### Collection of baseline features

Baseline characteristics comprised demographic factors, lifestyle factors, and health status. Demographic factors included age, gender (male or female), race/ethnicity (Mexican American, non-Hispanic Black, non-Hispanic White, other Hispanic, and other), educational level (less than high school, high school graduate, some college or associate degree, college or above), marital status (never married, widowed or divorced, married or living with partner), and family poverty-to-income ratio (0–1, 1–3, >3). Lifestyle factors encompassed moderate-to-vigorous physical activity (yes or no), alcohol consumption (never, light, moderate, or heavy), and smoking status (never, former, or now). Health status included BMI classification (normal, overweight, or obesity) and the presence of hypertension or hyperlipidemia. Data on age, gender, race/ethnicity, educational level, marital status, and family poverty-to-income ratio were obtained from the NHANES Demographic Data module, while information on alcohol consumption, smoking, and physical activity was sourced from the Questionnaire Data module. Alcohol consumption was categorized into four levels: heavy drinking (≥3 drinks per day for women and ≥4 drinks per day for men within the past 12 months), moderate drinking (2–3 drinks per day for women and 3–4 drinks per day for men within the past 12 months), light drinking (≤2 drinks per day for both men and women within the past 12 months), and never drinking (no alcohol consumption). Smoking status was classified as never smokers (fewer than 100 cigarettes smoked in their lifetime), former smokers (more than 100 cigarettes smoked but not currently smoking), and current smokers (more than 100 cigarettes smoked and currently smoking either occasionally or daily). BMI data were obtained from the Examination Data Module. Hypertension and hyperlipidemia were determined based on laboratory measurements and self-reported data. Hypertension was defined as systolic blood pressure (SBP) ≥ 130 mmHg and/or diastolic blood pressure (DBP) ≥ 80 mmHg on at least three occasions, or a self-reported history of hypertension diagnosis or antihypertensive medication use. Hyperlipidemia was defined as low high-density lipoprotein cholesterol (HDL-C) (<1.0 mmol/L for men and <1.3 mmol/L for women), triglycerides (TG) ≥ 1.8 mmol/L, or a self-reported history of hyperlipidemia diagnosis or lipid-lowering medication use. Chronic kidney disease (CKD) was defined according to established criteria as either an estimated glomerular filtration rate (eGFR) < 60 mL/min/1.73 m^2^ or a urine albumin-to-creatinine ratio (ACR) > 30 mg/g (27, 28).

### Pre-processing of machine learning features

The initial dataset included 57 features, comprising 46 continuous and 11 categorical variables. To mitigate multicollinearity among dietary antioxidants, we calculated the correlation coefficients and excluded features with a correlation coefficient exceeding 0.9. The data was then split into training and testing sets, and all features were standardized to eliminate scale differences. To address class imbalance, the Synthetic Minority Over-sampling Technique (SMOTE) was applied to the training set, generating synthetic samples and enhancing the model’s ability to learn from the minority class, while ensuring no data leakage into the testing set.

### Statistical analysis

This study employed a survey-weighted statistical model to characterize the comorbidity and non-comorbidity groups. Continuous variables were reported as mean ± standard deviation, while categorical variables were presented as frequencies and percentages. Group comparisons were performed using the weighted χ^2^ test for categorical variables, analysis of variance (ANOVA) for normally distributed continuous variables, and the Kruskal-Wallis *H* test for non-normally distributed variables.

This study implemented several machine learning models, including Recursive Partitioning and Regression Trees (RPART), Random Forest (RF), Kernel K-Nearest Neighbors (K-KNN), Naive Bayes (NB), Light Gradient Boosting Machine (LightGBM), Extreme Gradient Boosting (XGBoost), Multi-Layer Perceptron (MLP), and Support Vector Machine (SVM) using the mlr3 framework. RPART builds decision trees by recursively partitioning the data, effectively capturing nonlinear relationships and feature interactions, making it ideal for modeling complex variable dependencies (29). RF, as an ensemble method, mitigates overfitting by constructing multiple decision trees and averaging their predictions, handling intricate feature interactions (30). K-KNN classifies based on the similarity between samples, making it effective for nonlinear data, particularly when sample distribution is uneven or boundaries are unclear (31). NB relies on the naive Bayes assumption of feature conditional independence, offering high computational efficiency, particularly in high-dimensional, large-scale datasets (32). LightGBM, a gradient boosting tree algorithm, quickly builds efficient models on large datasets using efficient splitting strategies and parallel training while avoiding overfitting (33). XGBoost, based on gradient boosting optimization, offers robust regularization and excels at capturing complex nonlinear relationships, performing exceptionally well across diverse datasets (34). MLP uses multi-layer neural networks to capture intricate patterns and nonlinear relationships in input data, making it well-suited for complex tasks such as image and speech recognition (35). SVM identifies the optimal decision boundary by maximizing the margin between classes, making it effective for high-dimensional data and suitable for both linear and nonlinear problems, particularly in small sample, high-dimensional datasets (36). These models have been successfully applied in previous NHANES data analyses (37, 38), confirming their applicability.

Benchmarking is essential for evaluating and comparing ML model performance. This study assessed multiple models on a standardized dataset using consistent metrics to ensure fairness. For classification tasks, key evaluation metrics included classification error rate, accuracy, F-beta score, area under the ROC curve (AUC-ROC), sensitivity, specificity, and area under the PR curve (AUC-PR). AUC-ROC was the primary metric for performance assessment, while the other indicators provided a comprehensive evaluation of model effectiveness. To minimize evaluation bias, 10-fold cross-validation was employed for data resampling, and statistical differences across models were analyzed using analysis of variance (ANOVA) and the Kruskal-Wallis *H* test.

We utilized SHAP values to assess global feature importance in the best-performing ML model. Based on game theory, SHAP interprets the overall behavior of the model by aggregating the local contributions of each feature. It represents a state-of-the-art approach to interpretability for tree-based models. Compared to other global approximation methods, SHAP provides a more accurate measurement of feature impact on model decisions. In addition to offering a quantitative evaluation of overall feature importance, it also reveals the specific contribution of each feature to individual predictions, thus enhancing the model’s transparency and interpretability.

Data analysis was conducted using R statistical software (v4.4.1), with the following R packages: survey, DMwR, ggcor, mlr3, mlr3benchmark, mlr3extralearner, and shapviz. All statistical tests were two-sided, and a *p*-value of < 0.05 was considered statistically significant.

## Results

### Characteristics of the features

This study included a total of 1,356 participants, of whom 332 were diagnosed with both CVD and diabetes. Compared to diabetic participants without CVD, those with comorbid CVD had significantly lower intakes of Mg (281.08 ± 113.70 vs. 260.01 ± 109.17, *p* = 0.003), Se (107.99 ± 48.91 vs. 100.21 ± 41.89, *p* = 0.009), and Eriodictyol (0.15 ± 0.60 vs. 0.07 ± 0.18, *p* = 0.013). In addition, significant differences were observed between the two groups in demographic and clinical characteristics, including age, sex, race/ethnicity, education level, family income-to-poverty ratio, BMI, physical activity, smoking status, alcohol consumption, hypertension, hyperlipidemia and CKD (Table 1).

**Table 1:** Baseline characteristics of the participants.

	Overall	Diabetes without CVD	Diabetes with CVD	*p*-value
Participants	1,356	1,024	332	
Vitamin A (mcg)	602.79(534.73)	599.05(515.85)	614.34(589.85)	0.651
Vitamin C (mg)	78.18(71.35)	79.37(67.54)	74.52(82.01)	0.282
Vitamin E (mg)	7.24(4.54)	7.30(4.63)	7.08(4.25)	0.462
Mg (mg)	275.92(112.93)	281.08(113.70)	260.01(109.17)	0.003
Zinc (mg)	10.75(6.69)	10.85(7.13)	10.47(5.09)	0.368
Se (mcg)	106.08(47.39)	107.99(48.91)	100.21(41.89)	0.009
Carotenoid (mcg)	8952.88(9224.72)	9147.57(9465.32)	8352.39(8424.69)	0.172
Daidzein (mg)	0.39(1.86)	0.40(1.99)	0.35(1.42)	0.669
Genistein (mg)	0.53(2.67)	0.55(2.87)	0.47(1.91)	0.608
Glycitein (mg)	0.07(0.39)	0.07(0.42)	0.06(0.30)	0.637
Cyanidin (mg)	2.39(7.84)	2.33(7.87)	2.57(7.75)	0.62
Petunidin (mg)	0.87(3.42)	0.92(3.56)	0.73(2.92)	0.372
Delphinidin (mg)	1.23(4.63)	1.32(4.92)	0.97(3.56)	0.233
Malvidin (mg)	3.69(10.86)	3.83(11.35)	3.27(9.22)	0.414
Pelargonidin (mg)	1.12(3.82)	1.09(3.53)	1.22(4.61)	0.563
Peonidin (mg)	1.28(5.27)	1.20(4.98)	1.51(6.10)	0.356
Catechin (mg)	6.85(8.54)	6.86(7.82)	6.84(10.44)	0.968
Epigallocatechin (mg)	14.71(41.45)	14.17(32.77)	16.38(60.90)	0.397
Epicatechin (mg)	8.51(12.88)	8.40(10.70)	8.84(18.03)	0.588
Epicatechin 3-gallate (mg)	9.47(26.90)	9.11(21.84)	10.57(38.55)	0.391
Epigallocatechin 3-gallate (mg)	25.51(85.04)	24.00(59.70)	30.15(136.23)	0.253
Theaflavin (mg)	1.37(3.86)	1.39(3.91)	1.34(3.74)	0.854
Thearubigins (mg)	79.30(206.42)	80.22(209.93)	76.49(195.46)	0.775
Eriodictyol (mg)	0.13(0.53)	0.15(0.60)	0.07(0.18)	0.013
Hesperetin (mg)	8.77(17.29)	9.03(17.54)	7.95(16.52)	0.322
Naringenin (mg)	3.61(8.37)	3.73(8.68)	3.27(7.34)	0.393
Apigenin (mg)	0.18(0.39)	0.17(0.31)	0.19(0.55)	0.61
Luteolin (mg)	0.65(0.87)	0.67(0.89)	0.60(0.77)	0.192
Isorhamnetin (mg)	0.88(1.51)	0.88(1.40)	0.89(1.80)	0.916
Kaempferol (mg)	4.14(5.76)	4.20(5.50)	3.95(6.49)	0.485
Myricetin (mg)	1.37(2.26)	1.39(2.17)	1.31(2.52)	0.598
Quercetin (mg)	10.60(9.86)	10.71(9.74)	10.26(10.23)	0.474
Theaflavin 3,3′-digallate (mg)	1.52(4.27)	1.53(4.32)	1.47(4.12)	0.834
Theaflavin 3′-gallate (mg)	1.28(3.67)	1.29(3.71)	1.26(3.55)	0.897
Theaflavin 3-gallate (mg)	1.09(3.06)	1.10(3.10)	1.06(2.94)	0.799
Gallocatechin (mg)	1.46(3.80)	1.43(3.54)	1.54(4.50)	0.652
Subtotal Catechins (mg)	66.50(173.93)	63.97(131.21)	74.31(265.61)	0.347
Total Isoflavones (mg)	1.00(4.88)	1.03(5.23)	0.88(3.61)	0.631
Total Anthocyanidins (mg)	10.58(24.05)	10.68(24.62)	10.27(22.22)	0.788
Total Flavan-3-ols (mg)	151.07(356.03)	149.49(338.11)	155.93(406.87)	0.775
Total Flavanones (mg)	12.51(23.47)	12.91(23.90)	11.29(22.08)	0.275
Total Flavones (mg)	0.83(1.04)	0.84(1.02)	0.78(1.11)	0.371
Total Flavonols (mg)	16.99(17.13)	17.18(16.55)	16.41(18.83)	0.479
Total 29 Flavonoid (mg)	192.98(372.48)	192.14(354.47)	195.58(423.81)	0.884
Age	61.31(11.78)	59.90(12.04)	65.69(9.74)	<0.001
Gender				0.005
Female	816(60.18)	594(58.01)	222(66.87)	
Male	540(39.82)	430(41.99)	110(33.13)	
Race				<0.001
Mexican American	249(18.36)	210(20.51)	39(11.75)	
Non-Hispanic Black	142(10.47)	116(11.33)	26(7.83)	
Non-Hispanic White	519(38.27)	360(35.16)	159(47.89)	
Other Hispanic	344(25.37)	261(25.49)	83(25.00)	
Other Race-Including Multi-Racial	102(7.52)	77(7.52)	25(7.53)	
Education				0.043
Less than high school	433(31.93)	321(31.35)	112(33.73)	
High school graduate	331(24.41)	244(23.83)	87(26.20)	
Some college or associates degree	373(27.51)	277(27.05)	96(28.92)	
College or above	219(16.15)	182(17.77)	37(11.14)	
Marital Status				0.246
Never married	102(7.52)	82(8.01)	20(6.02)	
Widowed or divorced	428(31.56)	313(30.57)	115(34.64)	
Married or living with partner	826(60.91)	629(61.43)	197(59.34)	
FPIR level				0.036
> = 0, <=1	257(18.95)	195(19.04)	62(18.67)	
>1, <=3	626(46.17)	454(44.34)	172(51.81)	
>3	473(34.88)	375(36.62)	98(29.52)	
BMI	32.15(6.95)	31.87(6.62)	33.01(7.82)	0.009
Moderate to vigorous activity				0.001
No	512(37.76)	360(35.16)	152(45.78)	
Yes	844(62.24)	664(64.84)	180(54.22)	
Alcohol				<0.001
Never	491(36.21)	348(33.98)	143(43.07)	
Mild	526(38.79)	392(38.28)	134(40.36)	
Moderate	163(12.02)	140(13.67)	23(6.93)	
Heavy	176(12.98)	144(14.06)	32(9.64)	
Smoke				<0.001
Never	586(43.22)	473(46.19)	113(34.04)	
Former	518(38.20)	368(35.94)	150(45.18)	
Now	252(18.58)	183(17.87)	69(20.78)	
Hypertension				<0.001
No	330(24.34)	277(27.05)	53(15.96)	
Yes	1,026(75.66)	747(72.95)	279(84.04)	
Hyperlipidemia				0.002
No	105(7.74)	93(9.08)	12(3.61)	
Yes	1,251(92.26)	931(90.92)	320(96.39)	
CKD				<0.001
No	1,142(84.22)	901(87.99)	241(72.59)	
Yes	214(15.78)	123(12.01)	91(27.41)	

### Development and validation of the comorbidity disease prediction model

**Figure 2:**
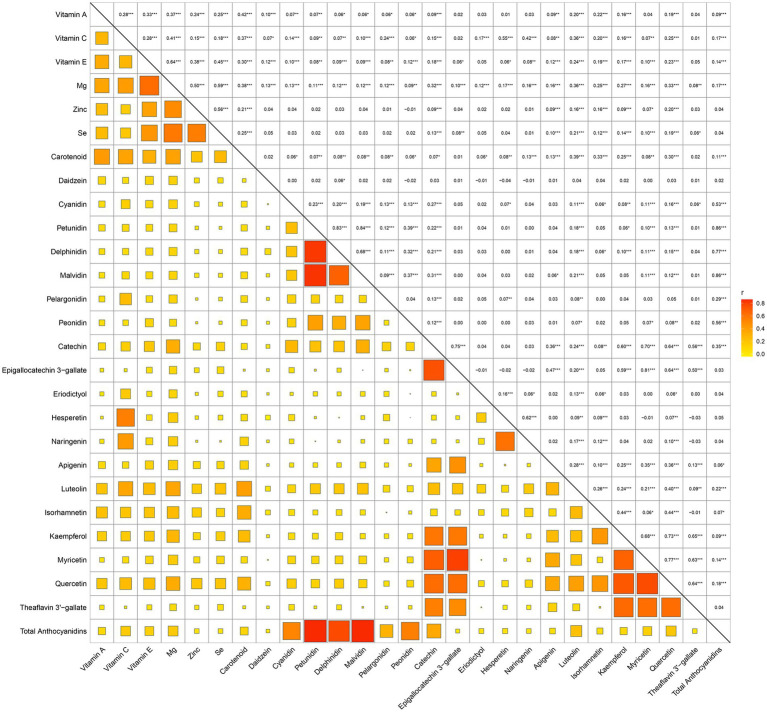
Correlation matrix of retained dietary antioxidant variables following collinearity exclusion.

Before constructing the ML model, we conducted a visual analysis of feature distributions. Correlation analysis of dietary antioxidants (Supplementary Figure 1) identified strong correlations among several features. Consequently, the following features were excluded: Genistein, Glycitein, Epigallocatechin, Epicatechin 3-gallate, Theaflavin, Thearubigins, Theaflavin 3,3′-digallate, Theaflavin 3-gallate and Gallocatechin. Figure 2 presents the dietary antioxidant features included in the ML model after addressing collinearity. In total, the model incorporated 27 dietary antioxidant features and 12 baseline features.

**Figure 3:**
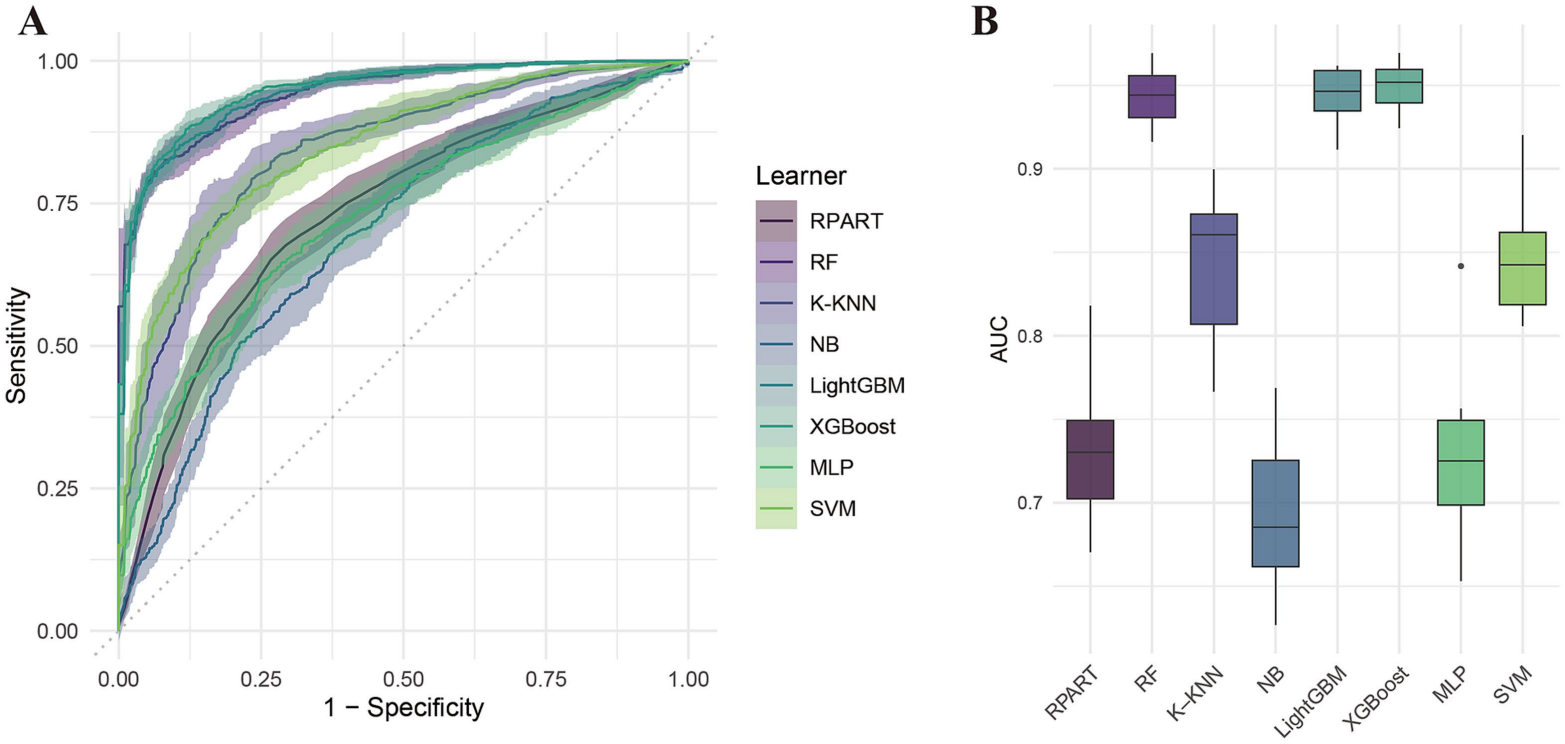
ROC curve analysis of eight machine learning models for predicting cardiovascular disease risk in diabetic patients. **(A)** ROC curves; **(B)** Area under the curve (AUC) comparison.

**Figure 4:**
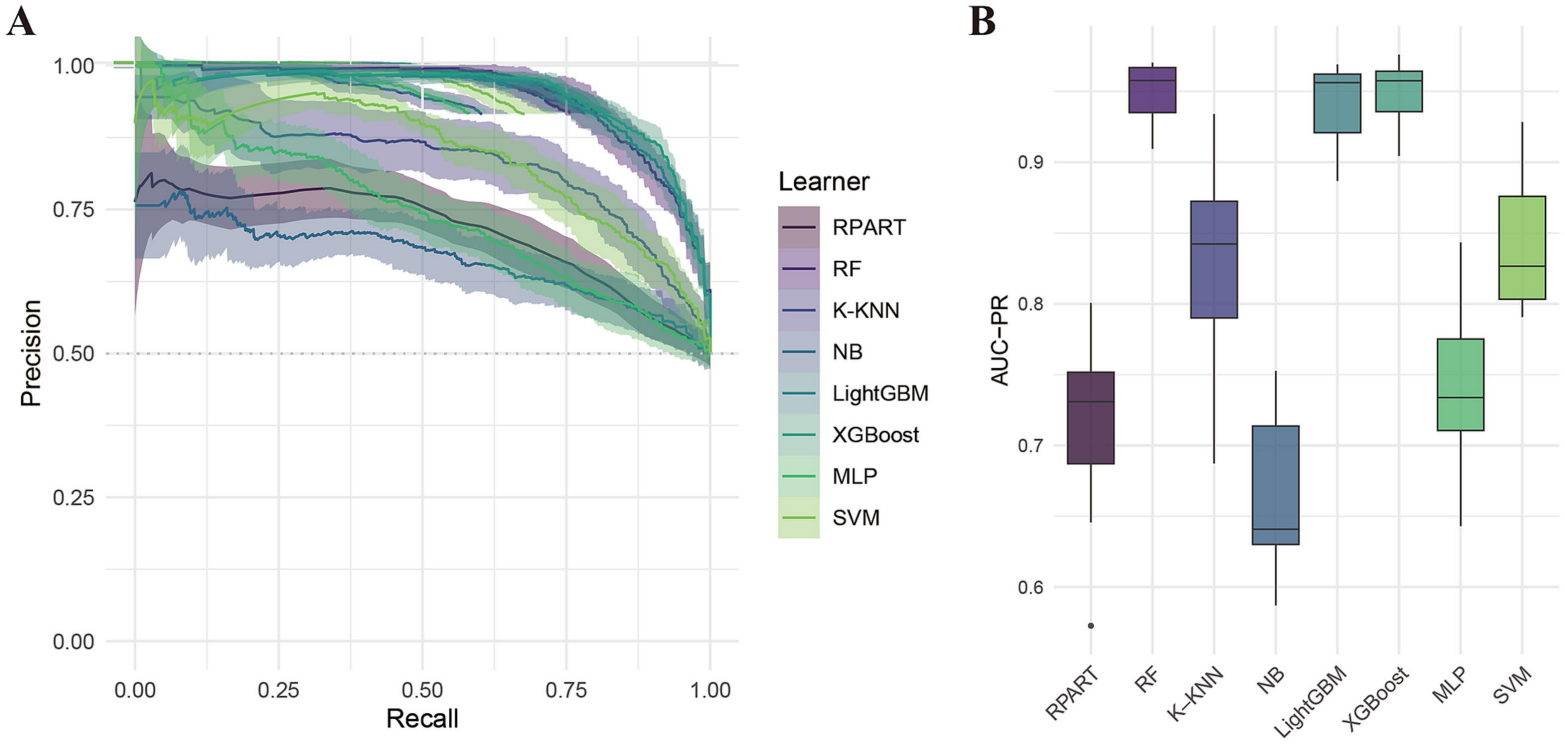
Precision-recall (PR) curve analysis of eight machine learning models. **(A)** PR curves; **(B)** Area under the PR curve (PR-AUC) comparison.

Table 2 summarizes the performance of eight machine learning models—RPART, RF, K-KNN, NB, LightGBM, XGBoost, MLP, and SVM—evaluated using key metrics including AUC (Figure 3), PR curve (Figure 4), classification error (Supplementary Figure 2), accuracy (Supplementary Figure 3), F-beta score (Supplementary Figure 4), sensitivity (Supplementary Figure 5), and specificity (Supplementary Figure 6). Among all the evaluated models, XGBoost demonstrated the highest overall performance, achieving an accuracy of 87.4% and the lowest classification error rate of 12.6%, indicating robust predictive accuracy and effective error control. The model also attained an area under the receiver operating characteristic (ROC) curve (AUC) and a precision-recall (PR) curve value of 0.949, highlighting its excellent classification capability and stable performance across varying precision-recall thresholds. LightGBM ranked second, with an accuracy of 86.3%, an AUC of 0.944, and a PR value of 0.942. RF followed closely, with an accuracy of 86.0%, an AUC of 0.944, and a PR value of 0.950. All three models exhibited sensitivity and specificity values approaching 90%, underscoring their high reliability and practical applicability. In contrast, K-KNN (accuracy: 77.8%), SVM (76.5%), RPART (68.6%), and MLP (67.4%) demonstrated moderate classification performance. K-KNN showed relatively high specificity (81.9%) but lower sensitivity (73.8%), whereas SVM had a sensitivity of 77.1% and a specificity of 75.7%. RPART presented moderate specificity (71.7%) but lower sensitivity (65.9%). MLP underperformed across both sensitivity and specificity, with both metrics below 70%. The NB model exhibited the poorest performance, with an accuracy of 60.9% and a high classification error rate of 39.1%. Despite its relatively high specificity (85.1%), it suffered from extremely low sensitivity (36.3%), limiting its utility in detecting true positive cases. Notably, the differences in key performance metrics across the models were statistically significant, indicating meaningful variability in predictive capabilities.

**Table 2:** Metrics of the eight machine learning models in predicting cardiovascular disease in diabetes.

Machine learner	Classification error rate	Accuracy	F-beta	Area under the ROC curve	Sensitivity	Specificity	Area under the PR curve
RPART	0.314	0.686	0.675	0.733	0.659	0.717	0.712
RF	0.140	0.860	0.858	0.944	0.858	0.862	0.950
K-KNN	0.222	0.778	0.767	0.843	0.738	0.819	0.83
NB	0.391	0.609	0.479	0.693	0.363	0.851	0.666
LightGBM	0.137	0.863	0.863	0.944	0.871	0.856	0.942
XGBoost	0.126	0.874	0.873	0.949	0.877	0.873	0.949
MLP	0.326	0.674	0.662	0.726	0.644	0.699	0.744
SVM	0.235	0.765	0.764	0.846	0.771	0.757	0.843
*p*-value	<0.001^b^	<0.001^b^	<0.001^a^	<0.001^b^	<0.001^b^	<0.001^a^	<0.001^a^

RPART: Recursive partitioning and regression trees; RF: Random Forest; K–KNN: Kernel k-Nearest Neighbors; NB: Naïve Bayes; LightGBM: Light Gradient Boosting Machine; XGBoost: Extreme Gradient Boosting; MLP - Multi-Layer Perceptron; SVM: Support Vector Machine. ^a^ANOVA test. ^b^Kruskal-Wallis.

### Importance of dietary antioxidant features interpreted by SHAP value

**Figure 5:**
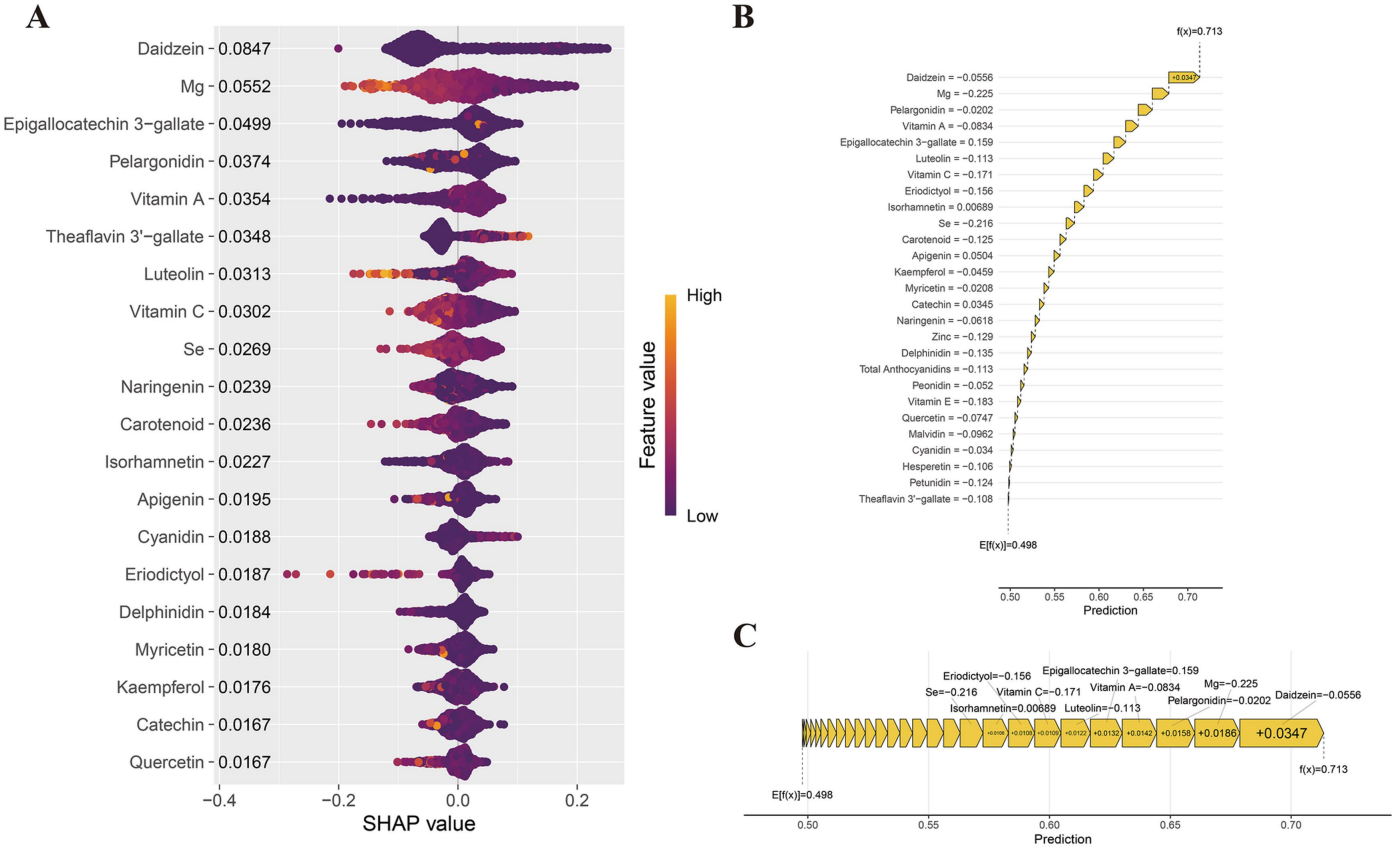
SHAP value interpretation of dietary antioxidant features in the XGBoost model. **(A)** SHAP summary plot; **(B)** SHAP waterfall plot; **(C)** SHAP force plot.

The SHAP analysis (Figure 5A and Supplementary Figure 7) identified the top 20 key dietary antioxidant features influencing comorbidity prediction. SHAP values highlighted Daidzein (0.085), Mg (0.055), EGCG (0.050), pelargonidin (0.037), vitamin A (0.035), and theaflavin 3′-gallate (0.035) as primary contributors. To visualize the impact of dietary antioxidants, we used the shapviz package to generate a waterfall plot (Figure 5B) and a force plot (Figure 5C). The waterfall plot illustrates each antioxidant’s contribution and cumulative effect on comorbidity prediction, with a final predicted probability of 0.713. Daidzein (−0.0556), Mg (−0.225), pelargonidin (−0.0202), vitamin A (−0.0834), and luteolin (−0.113) exhibited significant negative effects, suggesting that higher intake may reduce risk. The force plot (Figure 5C) highlights protective dietary antioxidants in yellow. Additionally, scatter plots in Supplementary Figure 8 show negative correlations between SHAP values and Vitamin E, Mg, Carotenoids, Daidzein, Malvidin, Pelargonidin, Epicatechin, Eriodictyol, Hesperetin, Luteolin, and Myricetin. These analyses provide insights into the model’s predictive logic, supporting personalized dietary recommendations.

## Discussion

This study, utilizing data from the 2007–2010 and 2017–2018 US NHANES cycles, systematically investigates the relationship between dietary antioxidant intake and CVD in diabetic patients through explainable machine learning techniques. Among the eight machine learning models developed, XGBoost demonstrated superior performance, achieving an average AUC of 0.955, highlighting its exceptional and consistent classification ability. The SHAP method, based on game theory, was employed to elucidate the contribution of each feature to the model’s predictions. The findings revealed that Daidzein, Mg, Isorhamnetin, Pelargonidin, Epigallocatechin 3-gallate, and Se were key influencing factors. To the best of our knowledge, this study is the first to integrate various antioxidants with baseline characteristics to build and validate a model for predicting CVD risk in diabetic patients. While the focus is on dietary antioxidants, the model also incorporates traditional features such as demographic factors, lifestyle, and health status, significantly enhancing prediction accuracy. Furthermore, systematic benchmarking was conducted to ensure a fair comparison and robust results across different models.

ML models have been increasingly utilized to explore dietary factors associated with cardiovascular disease. For instance, Ravi V. Shah and colleagues, using data from 2,259 young white and black adults in the CARDIA cohort, employed multivariate analysis and penalized machine learning techniques to identify metabolite features linked to dietary intake, encompassing 17 food groups, 2 nutrient types, and the Healthy Eating Index (HEI-2015). Their study highlighted that metabolic features associated with unhealthy dietary patterns, such as high intake of red/processed meat and refined grains, were stronger predictors of long-term diabetes and cardiovascular disease risks than traditional dietary scores (39). Similarly, Orly Ben-Yacov and colleagues applied machine learning algorithms to evaluate the effects of personalized postprandial target (PPT) diets compared to the Mediterranean diet in adults with prediabetes, showing that the PPT diet significantly improved cardiometabolic markers by modulating the gut microbiota, emphasizing the value of personalized nutrition strategies (40). Yue Wang and colleagues analyzed data from 90,167 participants in the UK Biobank using four machine learning algorithms, with the XGBoost model revealing that potassium, vitamin E, and vitamin C were significant predictors of CVD risk (41). Subgroup analyses identified calcium intake as a key factor for CVD risk in older adults and those with high BMI, while vitamin B6 was closely linked to CVD risk in women (41). Agustin Martin-Morales and colleagues compared multiple ML models, including logistic regression, support vector machines, RF, XGBoost, and LightGBM, selecting RF as the most effective model. SHAP analysis was used to identify critical factors such as age, systolic blood pressure, fiber, calcium, and vitamin E in predicting cardiovascular mortality (42). These studies illustrate the application of ML in examining the link between dietary factors and disease, offering valuable insights for the fields of cardiovascular disease in diabetes. However, many of these studies have limitations, such as the lack of systematic comparisons of model performance and benchmark evaluations, despite utilizing multiple machine learning models.

We selected several models, including RPART, RF, K-KNN, NB, LightGBM, XGBoost, MLP, and SVM, to develop a prediction system for cardiovascular disease in diabetes, and assessed each model’s performance through benchmarking to identify the most suitable approach. Compared to traditional statistical methods, such as logistic regression, machine learning offers notable advantages. First, machine learning is capable of capturing complex nonlinear relationships, whereas traditional methods typically assume linearity, limiting their effectiveness in addressing complex issues. Second, machine learning can automatically identify and select key predictive features, eliminating the subjective and labor-intensive feature selection required in traditional methods. Additionally, machine learning makes fewer assumptions about data distribution and can handle numerical and categorical data, as well as missing values, unlike traditional methods, which often rely on strict distribution assumptions. Machine learning, particularly with ensemble techniques like random forests and XGBoost, demonstrates robust predictive power by effectively preventing overfitting and improving model generalization. Thus, machine learning is more adaptable and efficient for handling complex datasets, offering more accurate decision-making support for disease prediction and personalized healthcare.

Our results demonstrate that the XGBoost model exhibited superior performance among the machine learning algorithms evaluated. As an advanced gradient boosting technique, XGBoost provides distinct advantages in processing complex, high-dimensional datasets. By aggregating multiple weak learners through decision trees and incorporating key strategies such as regularization, column sampling, and gradient-based optimization, XGBoost achieves both high predictive accuracy and strong generalization ability. Its inherent resistance to overfitting further ensures reliable performance across diverse datasets. Owing to these strengths, XGBoost has been extensively applied in the analysis of electronic health records for the development of robust disease prediction models (43–45).

In this study, dietary antioxidants are categorized into three primary types: vitamins, minerals, and polyphenols, each exerting antioxidant effects through distinct mechanisms. Vitamin C directly scavenges free radicals, regenerates oxidized vitamin E, and inhibits metal ion-induced oxidation reactions, thereby protecting cells from oxidative damage (46). Vitamin E, embedded in cell membranes, prevents lipid peroxidation by halting free radical chain reactions, thus preserving membrane integrity (47). Vitamin A and *β*-carotene effectively neutralize singlet oxygen, safeguarding epithelial cells and preventing lipid oxidation (48). Selenium, as a crucial component of glutathione peroxidase (GPx), reduces the harmful effects of hydrogen peroxide and lipid peroxides, while also synergizing with vitamin E to enhance antioxidant defense (49). Zinc scavenges free radicals by promoting metallothionein expression and serving as a cofactor for superoxide dismutase (Cu/Zn-SOD), thereby maintaining cellular redox balance and stabilizing cell membranes to protect against oxidative damage (50). Magnesium mitigates oxidative stress-induced cellular damage by regulating redox balance, supporting ATP synthesis, and maintaining mitochondrial function (51). Dietary polyphenols, known for their potent antioxidant properties, neutralize reactive oxygen species (ROS) and reactive nitrogen species (RNS) such as superoxide anions (O₂•^−^), hydroxyl radicals (•OH), and hydrogen peroxide (H₂O₂) through their phenolic hydroxyl groups (52). Additionally, polyphenols activate the Nrf2 pathway to increase the expression of endogenous antioxidant enzymes like superoxide dismutase (SOD), glutathione peroxidase (GPx), and glutathione (GSH) (53–55), while reducing chronic inflammation by modulating inflammatory pathways such as NF-κB (56), thereby offering combined antioxidant and anti-inflammatory effects.

Daidzein and Mg are two of the most critical antioxidants examined in this study. Daidzein, an isoflavone primarily found in soy and its derivatives, including tofu, soy milk, soybeans, and bean sprouts, has been clinically confirmed for its potential role in the prevention and treatment of cardiovascular diseases. For example, a cross-sectional study by D. Goodman-Gruen et al. demonstrated that postmenopausal women who consumed high amounts of soy isoflavones, such as Genistein, had significantly lower body mass index (BMI), waist circumference, and fasting insulin levels compared to those who did not consume isoflavones (57). Additionally, isoflavone intake was positively correlated with high-density lipoprotein cholesterol (HDL-C) levels and negatively correlated with postprandial insulin levels, suggesting that dietary soy may have protective effects on cardiovascular health in postmenopausal women (57). In a 16-week randomized controlled trial, Lea Tischmann et al. observed that soy nuts reduced low-density lipoprotein cholesterol (LDL-C) and mean arterial pressure (MAP), while significantly improving endothelial function in healthy elderly individuals (58). In a randomized crossover trial, K.E. Wangen et al. found that a high soy isoflavone diet significantly lowered LDL cholesterol and the LDL/HDL cholesterol ratio in postmenopausal women, indicating potential benefits in improving lipid profiles and reducing the risk of coronary heart disease (59). Moreover, a meta-analysis revealed that isoflavone intake significantly reduced triglyceride (TG) levels and moderately increased HDL-C levels in postmenopausal women, with more pronounced effects observed in women under the age of 65 (60). Another meta-analysis involving 2,305 postmenopausal women showed that soy protein containing isoflavones and soy isoflavone extracts significantly reduced total cholesterol and triglyceride levels while moderately increasing HDL-C, further supporting the potential benefits of soy-based products in improving lipid metabolism and reducing cardiovascular risk (61). Similarly, Daidzein has demonstrated potential cardiovascular benefits in several preclinical studies. Its mechanisms of action include antioxidant properties that reduce free radical generation and alleviate oxidative stress, thereby mitigating endothelial cell damage and lowering the risk of atherosclerosis (62, 63). Moreover, Daidzein plays a role in regulating lipid metabolism by decreasing total cholesterol, LDL-C, and triglyceride levels, while simultaneously increasing HDL-C levels, leading to improved lipid profiles (64). Additionally, Daidzein has anti-inflammatory effects, inhibiting the expression of pro-inflammatory factors, which contributes to enhanced vascular health (65). It also promotes the synthesis of nitric oxide (NO), which enhances endothelial function, improves vasodilation, and supports vascular elasticity, ultimately aiding in blood pressure regulation and improving blood flow (66). Furthermore, Daidzein exhibits antithrombotic properties by reducing platelet aggregation and enhancing fibrinolytic activity, thus lowering the risk of thrombosis (62). Mg ranks second in importance according to SHAP values. As the most abundant divalent cation in cells, Mg is essential for maintaining cellular physiological functions and metabolism. It acts as a cofactor for numerous enzymes, regulates ion channels, and supports energy production (67). In the cardiovascular system, Mg plays a critical role in neuronal excitability, cardiac conduction, and myocardial contraction by modulating ion transport proteins, such as potassium and calcium channels (67, 68). Research has shown that low serum magnesium levels or inadequate dietary intake are closely linked to an increased risk of hypertension (69), atherosclerosis (70), coronary artery disease (71), arrhythmias (72), and heart failure (73).

Our findings also suggest that dietary antioxidants, including EGCG, pelargonidin, vitamin A, and theaflavin 3′-gallate, play crucial roles in CVD prevention through distinct mechanisms. EGCG demonstrates potent ROS scavenging, metal ion chelation, inhibition of lipid peroxidation and oxidative enzymes, and activation of the Nrf2-ARE pathway, thereby enhancing cellular antioxidant defenses (74). It also reduces atherosclerosis risk by improving endothelial function, lowering inflammatory cytokines, and regulating blood pressure and lipid levels (74, 75). Pelargonidin, through its phenolic hydroxyl groups, scavenges free radicals, alleviates oxidative stress, reduces lipid accumulation, and enhances lipid profiles and endothelial function, thus decelerating atherosclerosis progression (76, 77). Vitamin A, a vital fat-soluble antioxidant, stabilizes cell membranes, modulates gene expression linked to endothelial repair and inflammation, and inhibits arterial remodeling and vascular aging via nuclear receptor mechanisms (78). Theaflavin 3′-gallate effectively prevents LDL oxidation, reduces vascular inflammation, and exhibits anti-platelet, lipid-lowering, and antihypertensive effects, thus disrupting multiple cardiovascular risk pathways (79, 80). These natural bioactive compounds offer multi-targeted antioxidant, anti-inflammatory, lipid-regulating, and vascular-protective effects, providing a comprehensive approach to CVD prevention.

Our study holds certain clinical application value. Firstly, the developed predictive model demonstrated strong performance in assessing CVD risk, indicating the potential for future non-invasive risk stratification in diabetic patients through dietary intake assessments. Secondly, this study identifies a significant association between several antioxidant nutrients and CVD risk in diabetic patients, providing evidence to inform clinical dietary recommendations. Consistent with the guidelines from the American Diabetes Association (ADA) and other relevant nutritional frameworks, the findings offer specific guidance for dietary interventions targeting CVD risk in diabetic individuals. The study highlights that antioxidants such as soy isoflavones (e.g., Daidzein), Mg, isorhamnetin, pelargonidin, epigallocatechin gallate (EGCG), and Se are strongly correlated with CVD risk. Consequently, it is recommended that diabetic patients incorporate moderate amounts of soy products (such as soy milk and tofu) to achieve a daily intake of 25–50 mg of isoflavones; consume 310–420 mg of magnesium daily from sources like leafy vegetables, nuts, and whole grains; increase the consumption of fruits and vegetables rich in isorhamnetin and pelargonidin (e.g., apples, onions, and berries), aiming for at least 400 grams per day; drink 1–2 cups of green tea per day to supplement EGCG; and ensure an intake of approximately 55 μg of selenium, primarily from natural sources like Brazil nuts and seafood. By optimizing the intake of these antioxidant nutrients, diabetic patients may further reduce their CVD risk in addition to blood glucose control. Lastly, although various small-molecule antioxidants have shown promise in preclinical research, clinical trials have yielded inconsistent or unsatisfactory outcomes. Our findings may offer valuable insights for future mechanistic studies and the refinement of evidence-based nutritional intervention strategies.

This study has several limitations. First, the diagnosis of diabetes and cardiovascular disease was partially based on self-reported data from the NHANES interview questionnaire, which may introduce information bias due to recall bias or cognitive limitations. Second, variations in dietary habits across different populations and regions could influence the model’s predictions, but further analysis was not possible due to the lack of relevant data. As cross-sectional data were used, this study is unable to establish causal relationships, and future longitudinal studies will be necessary to validate the model’s effectiveness. Although the use of the nationally representative NHANES dataset, along with the inclusion of factors such as gender, race, income, lifestyle, and health status, enhances the generalizability of the results, differences in dietary habits and health conditions across countries and regions may limit the external validity of the findings. Future research should aim to validate the model in diverse countries and dietary contexts. In addition, the complexity and limited interpretability of the model may impact its reproducibility and practical utility. Although SHAP values facilitate the assessment of feature contributions, they rely on the assumption of feature independence and may be affected by residual inter-feature correlations. While highly collinear variables were excluded in this study, the interpretation of feature importance should be approached with caution. Future efforts toward more rigorous feature selection may improve model robustness, though this must be balanced against potential information loss. Finally, the study observed a significant age difference between the DM without CVD group and the DM + CVD group, as well as a higher proportion of females in the CVD group. These factors may influence the results. While machine learning methods can partially adjust for these differences, future research should conduct more detailed analyses of age, gender, and other potential confounders, and use more representative samples to minimize bias.

## Conclusion

In conclusion, we developed and validated a cardiovascular disease prediction model for diabetic patients using eight different algorithms: RPART, RF, K-KNN, NB, LightGBM, XGBoost, MLP, and SVM. Of these, XGBoost exhibited the highest discrimination and accuracy in predicting cardiovascular disease in diabetes. SHAP value analysis further elucidated the roles and contributions of various antioxidants, with Daidzein and Mg emerging as the key antioxidants in the model.

## Data Availability

The original contributions presented in the study are included in the article/Supplementary material, further inquiries can be directed to the corresponding author.

## Glossary

CVD: cardiovascular disease
NHANES: National Health and nutrition examination survey
RPART: recursive partitioning and regression trees
RF: random ForestRF
K-KNN: kernel K-nearest neighbors
NB: Naive Bayes
LightGBM: light gradient boosting machine
XGBoost: extreme gradient boosting
MLP: multi-layer perceptron
SVM: support vector machine
SHAP: SHapley additive explanation
Mg: magnesium
EGCG: epigallocatechin-3-gallate
Se: selenium
DM: diabetes mellitus
AGEs: advanced glycation end products
MI: myocardial infarction
ML: machine learning
SMOTE: synthetic minority over-sampling technique
ROC: receiver operating characteristic
PR: precision-recall
AUC-ROC: area under the ROC curve
AUC-PR: area under the PR curve
PPT: personalized postprandial target
GPx: glutathione peroxidase
ROS: reactive oxygen species
RNS: reactive nitrogen species
SOD: superoxide dismutase
GSH: glutathione
BMI: body mass index
HDL-C: high-density lipoprotein cholesterol
LDL-C: low-density lipoprotein cholesterol
MAP: mean arterial pressure
TG: triglyceride
NO: nitric oxide
