# Supplementary material for: Machine learning and SHAP value interpretation for predicting cardiovascular disease risk in patients with diabetes using dietary antioxidants
Source: Front Nutr. 2025 Jul 16;12:1612369. doi: 10.3389/fnut.2025.1612369 (PMC12307172; doi:10.3389/fnut.2025.1612369)

Supplemental Figures

Supplementary Figure 1. Heatmap showing correlations among dietary antioxidant variables.
Supplementary Figure 2. Classification error rates of eight machine learning models for CVD risk prediction in diabetic patients.
Supplementary Figure 3. Prediction accuracy comparison across eight machine learning models.
Supplementary Figure 4. F-beta score comparison across eight machine learning models.
Supplementary Figure 5. Sensitivity comparison across eight machine learning models.
Supplementary Figure 6. Specificity comparison across eight machine learning models.
Supplementary Figure 7. Top 15 dietary antioxidants ranked by mean SHAP values in the XGBoost model.
Supplementary Figure 8. SHAP value scatter plots illustrating associations with dietary antioxidant intake levels in the XGBoost model.

Supplementary Figure 1. Heatmap showing correlations among dietary antioxidant variables.

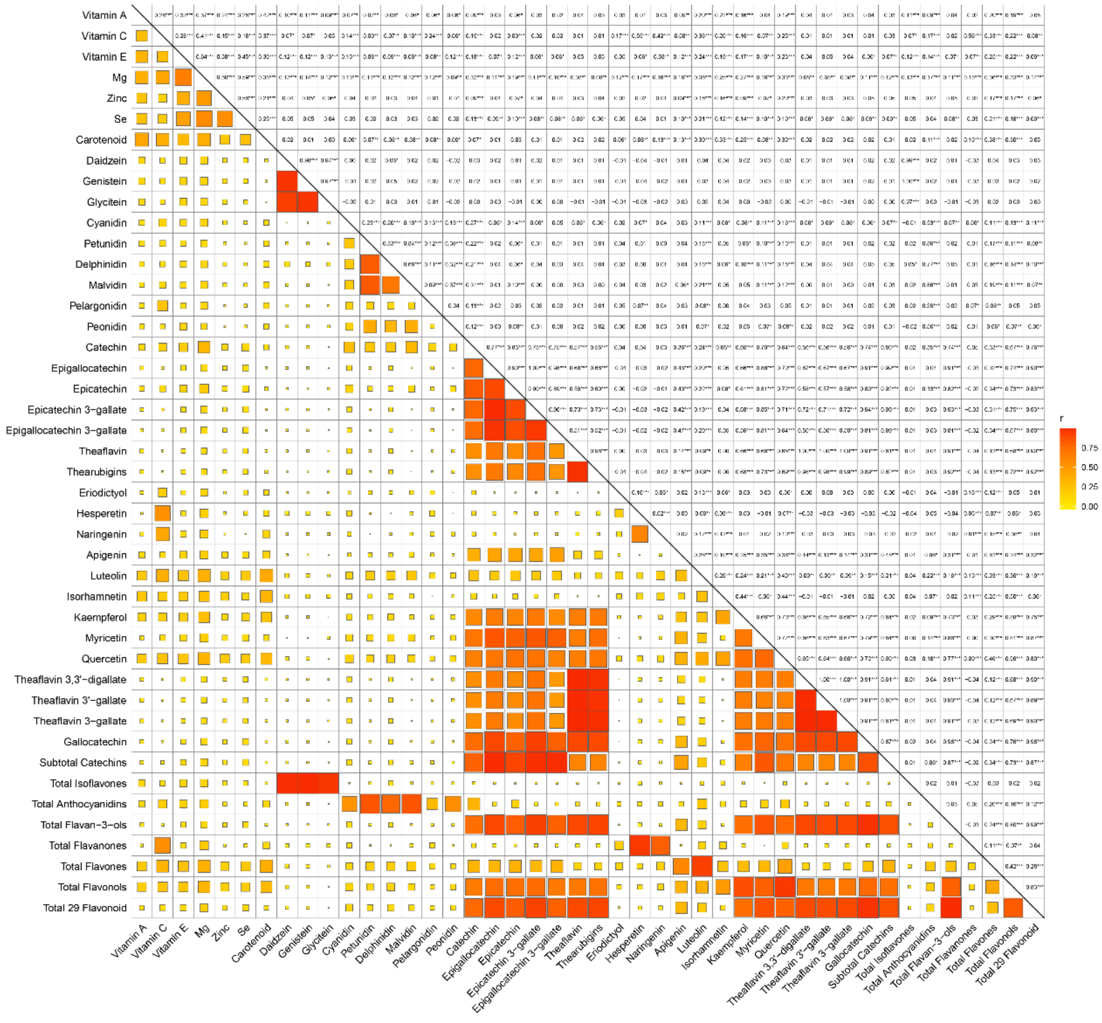

Supplementary Figure 2. Classification error rates of eight machine learning models for CVD risk prediction in diabetic patients.

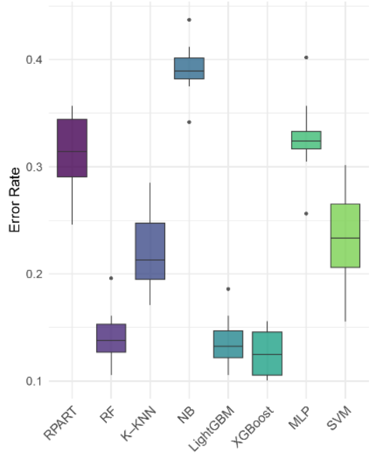

Supplementary Figure 3. Prediction accuracy comparison across eight machine learning models.

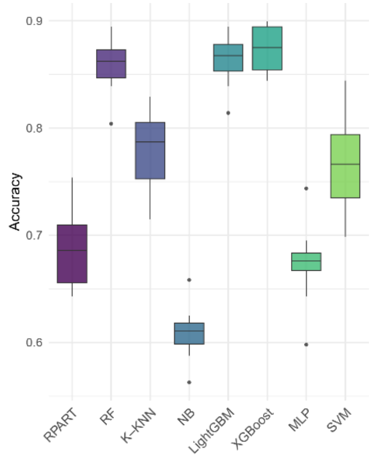

Supplementary Figure 4. F-beta score comparison across eight machine learning models.

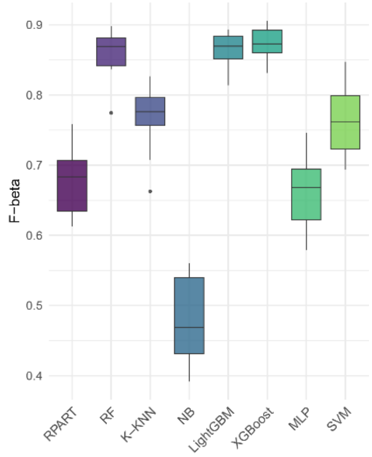

Supplementary Figure 5. Sensitivity comparison across eight machine learning models.

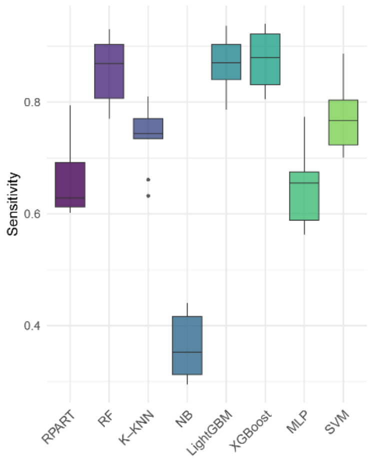

Supplementary Figure 6. Specificity comparison across eight machine learning models.

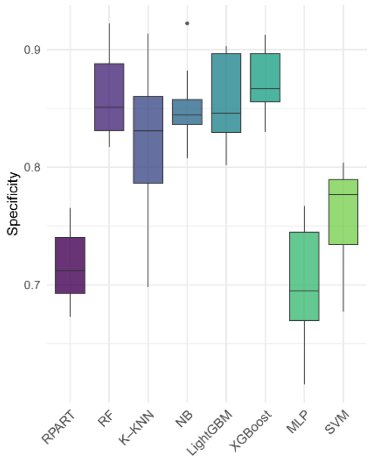

Supplementary Figure 7. Top 15 dietary antioxidants ranked by mean SHAP values in the XGBoost model.

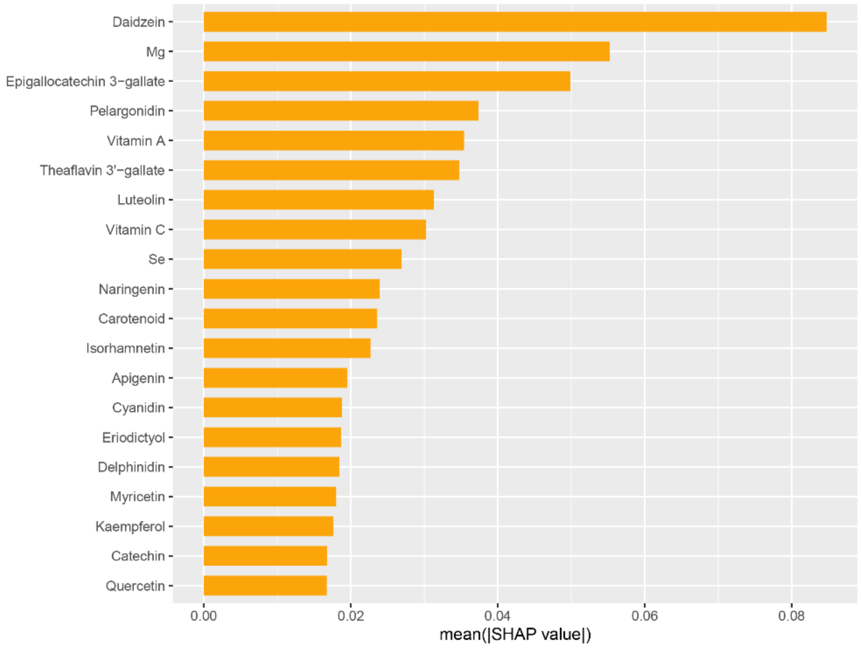

Supplementary Figure 8. SHAP value scatter plots illustrating associations with dietary antioxidant intake levels in the XGBoost model.

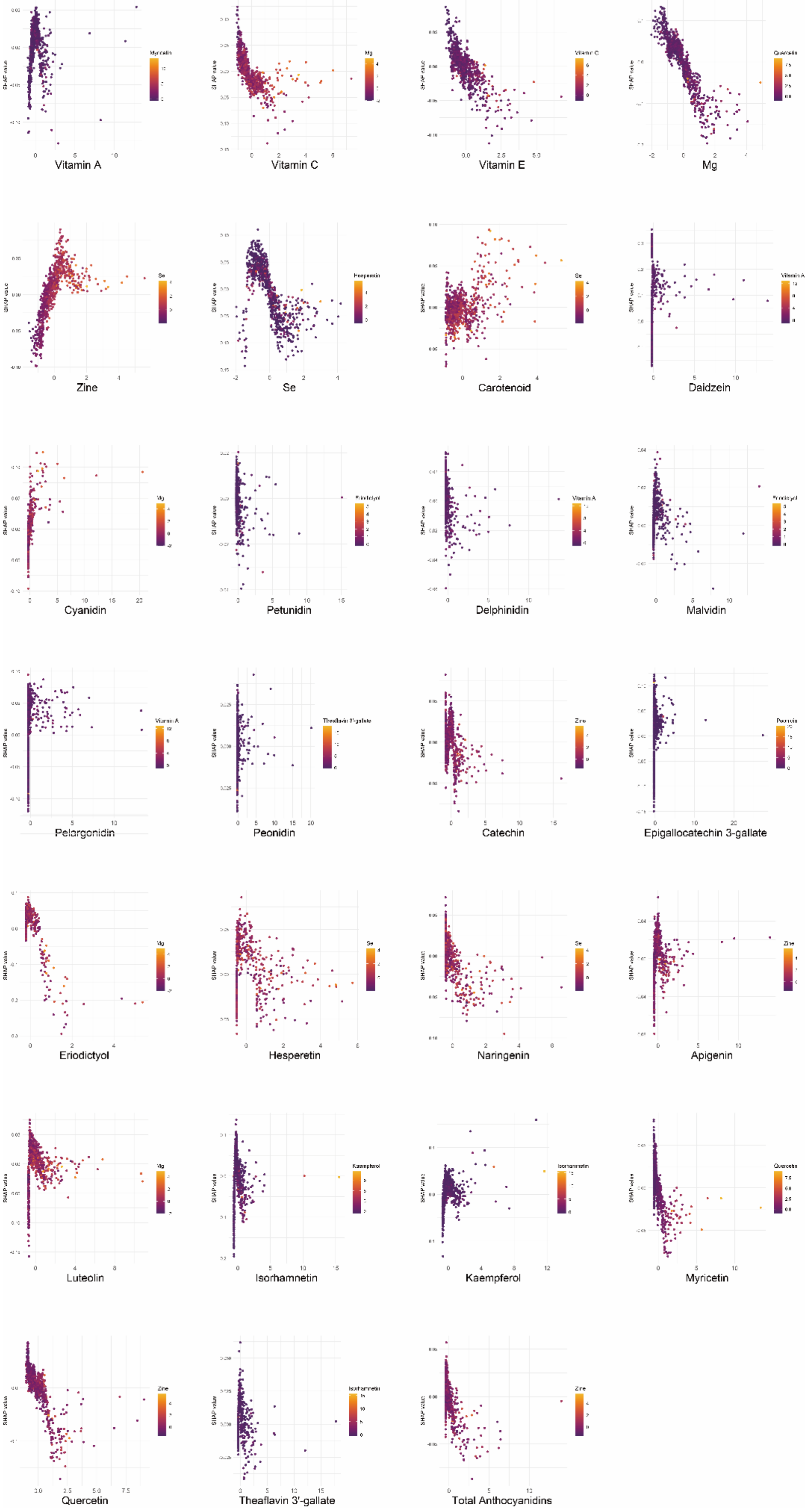

Supplement: Supplementary file 1 [file Image_1.pdf]
